# Differences in a Cage Escape Behaviour between Two Migrating Warblers of Different Stop-Over Strategy

**DOI:** 10.3390/ani11030639

**Published:** 2021-02-28

**Authors:** Dariusz Jakubas, Katarzyna Wojczulanis-Jakubas, Alexis Powers, Troy Frazier, Michael Bottomley, Michał Kraszpulski

**Affiliations:** 1 Department of Vertebrate Ecology and Zoology, Faculty of Biology, University of Gdańsk, 80-308 Gdańsk, Poland; biokwj@univ.gda.pl; 2 Departments of Biology, Wright State University, Dayton, OH 45435, USA; powers.470@buckeyemail.osu.edu; 3 Psychology, Wright State University, Dayton, OH 45435, USA; frazier.101@wright.edu (T.F.); michal.kraszpulski@wright.edu (M.K.); 4 Mathematics and Statistics, Wright State University, Dayton, OH 45435, USA; michael.bottomley@wright.edu; 5 NCBP, Wright State University, Dayton, OH 45435, USA

**Keywords:** cognitive abilities, reed and Sedge Warblers, migratory birds, stop-over ecology

## Abstract

**Simple Summary:**

Cognitive abilities play an important role for migratory birds that are briefly visiting a variety of unfamiliar stop-over habitats. Here, we compared cognitive abilities-linked behaviour (escape from an experimental cage) between two long-distant migrants differing in refuelling strategy during autumn migration, Sedge Warbler (not territorial, searching for locally superabundant food) and Reed Warbler (territorial, foraging on a common prey). We performed a cage experiment on individuals captured in mist-nets. After two minutes of acclimatization in the cage, we remotely opened the cage door and recorded the bird’s reaction. We measured latency that individuals needed to escape from a cage. Sedge warblers were more likely to escape from the cage than Reed Warblers. Sedge warblers generally escaped earlier after the door was opened and were more likely to escape at any given time than Reed Warblers. We interpret the prevalence of non-escaped individuals as a general feature of migratory birds. In contrast to resident species, they are more likely to enter an unfamiliar environment, but they are less explorative. Differences in escape latency between the studied species may be linked to various refuelling strategies in the context of specialist-generalist foraging. Our study provides ecological insight into the cognitive abilities-linked behaviour of wild animals.

**Abstract:**

Cognitive abilities play an important role for migratory birds that are briefly visiting a variety of unfamiliar stop-over habitats. Here, we compared cognitive abilities-linked behaviour (escape from an experimental cage) between two long-distant migrants differing in stop-over ecology, Sedge Warbler (*Acrocephalus schoenobaenus*; not territorial, searching for locally superabundant food) and Reed Warbler (*A. scirpaceus*; territorial, foraging on a common prey) during the autumn migration. After two minutes of acclimatization in the cage, we remotely opened the cage door and recorded the bird’s reaction. We measured latency that individuals needed to escape from a cage. Sedge warblers were 1.61 times more likely to escape from the cage than Reed Warblers. Sedge warblers generally escaped earlier after the door was opened and were 1.79 times more likely to escape at any given time than Reed Warblers. We interpret the prevalence of non-escaped individuals as a general feature of migratory birds. In contrast to resident species, they are more likely to enter an unfamiliar environment, but they are less explorative. We attributed inter-species differences in escape latency to species-specific autumn stop-over refuelling strategies in the context of specialist-generalist foraging. Our study provides ecological insight into the cognitive abilities-linked behaviour of wild animals.

## 1. Introduction

Cognitive abilities have been recently recognized to be an important component of animal adaptation to the changing environment. Numerous studies have demonstrated that animals actively acquire, processes, and store environmental or social information to use in the more or less distant future (reviewed in [1,2,3]). However, despite a large number of evidences on animal cognition *in sensu lato*, we are still far from recognizing the cognitive performance across animal taxa, which in turn inhibits our understanding of the mechanisms behind adaptivity of cognitive abilities.

Migratory birds evolved to perform seasonal migrations by the development of various adaptive traits, such as morphological (e.g., pointed wings), physiological (e.g., pre-migratory fattening), hormonal (e.g., lower melatonin production), neurological (detection of magnetic field), and behavioural ones (e.g., migratory restlessness), widely studied by researchers [4,5]. Nevertheless, cognitive abilities of migratory birds are less frequently studied compared to the aforementioned traits [6] while the group is particularly interesting in that context. Seasonal migration with an imposed change of the habitat requires a long-lasting memory and fast acquisition to unfamiliar environments, clearly cognitive abilities-linked traits. Therefore, investigating cognitive performance in wild migratory species may provide new insight into animal cognition.

**Figure 1:**
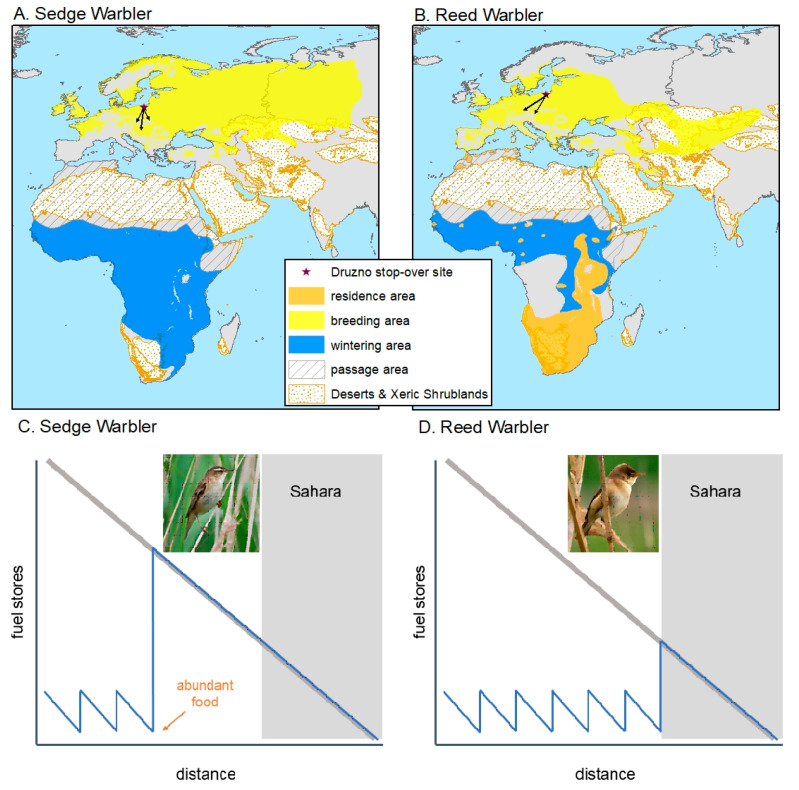
Breeding, wintering and passage areas of the sedge (**A**) and Reed Warblers (**B**) [17]. The range of Sahara desert habitats (biomes Deserts & Xeric Shrubland and Flooded Grasslands & Savannas) is according to Ecoregions2017© ^Resolve^ [18]. Summary of the refuelling strategies along Atlantic migration flyway from the breeding grounds in Europe to sub-Saharan Africa in terms of fuel stores for Sedge Warbler [(**C**); start of non-refuelling migration when encountering superabundant food, well before the Sahara (grey box)] and Reed Warbler [(**D**); large fuel deposition just before the Sahara (grey box)] according to [14]. The diagonal grey bar-line indicates the fuel load needed for flying to the southern border of the Sahara (grey area) without refuelling. Main migration directions of sedge and Reed Warbler populations (black arrows) migrating through the stop-over site in “Druzno Lake” reserve (black star) based on data from ringing recoveries from this site (after [19]).

In this study we examined a cage escape behaviour of two wild migratory avian species. Specifically, we measured an individual’s ability to adjust to a novel condition (here placement in an experimental cage), to perceive a change in environment (cage gate opening) and process and use this information (finding it as an opportunity to escape and making decision about escaping). Measured this way, the cage escaping behaviour is expected to be strongly linked to individual cognitive abilities [1]. We compared the cage escape behaviour between Sedge Warblers (*Acrocephalus schoenobaenus*) and Reed Warblers (*A. scirpaceus*). The two species are closely related passerines, both long-distant migrants, and insectivores of similar morphology. Both breed in Eurasia and winter in Central Africa [7], follow the same migration routes and use similar stop-over habitats. There are, however, some stark inter-species differences in migration strategy (Figure 1). Sedge warblers in autumn tend to fuel up extensively as soon as they encounter a site with super-abundant food (aphids or other aggregated prey [8,9,10,11,12,13]) and then fly directly to wintering grounds without refuelling [14,15,16]. Reed warblers, in contrast, do not fly directly from Central Europe to its wintering areas; they accumulate at each stopover site the amount of energy which is needed to fly safely to the next one. Besides, Reed Warblers were reported to defend small temporary territories at some stopover sites to secure successful fuelling ([8]; Walinder et al. in [15]) while Sedge Warblers, in contrast, are not territorial at stopover sites [8]. All these behavioural differences between the two species during their migration are likely to impose the difference in the cognitive abilities-linked traits.

Although studies on the cognitive abilities-linked traits of migratory birds are indeed scarce, existing ones suggest that migrants exhibit quite strong reliance on memory, low spatial neophobia but also low propensity to explore a new environment. This is in contrast to resident or even partial migrants and nomadic species, which all exhibit more explorative behaviour in their familiar environment [6,20,21]. Difference in cognitive performance-linked traits are also detectable at the intra-specific level. In a partially migratory species (blue tit, *Cyanistes caeruleus*), migrating individuals have shorter approach latencies to novelty compared to resident ones [22]. Thus, when examining the two warbler species in the present study we expected that Reed Warblers, being more territorial at the stop-over sites [8], should act more like a resident species (i.e., be more attentive to changes in the environment than Sedge Warblers). We do not have direct evidence for similarity of behaviour of resident and territorial species, however, we assume that both groups should be familiarized well with the core area where they spend most of their time. In contrast, non-territorial species exploring wider area would have more superficial knowledge of the surroundings. As such we expected Reed Warblers to be more responsive to the experimental set up (i.e., escaping the open cage more frequently with shorter escape latency than Sedge Warblers).

## 2. Materials and Methods

### 2.1. Study Area

We performed the study in the southern part of the Lake Druzno reserve (54°05′ N, 19°27′ E) in northern Poland, during the annual autumn trapping of small passerines, carried out there regularly since 1990. Lake Druzno is a large, shallow eutrophic lake, overgrown for much of its area with reedbeds, which makes it an attractive place for breeding and migrating reed and Sedge Warblers and other reedbeds-associated passerines [23,24]. We captured birds in mist-nets, with no playback, according to a daily schedule from dawn to dusk. We identified, aged, and ringed all captured birds.

### 2.2. Cage Escape Experiment

We performed cage experiments in August (core of the migration period at the study site [11,25,26]) in 2016, 2017, and 2019; thus, birds captured at that time represent mainly migratory individuals [23,27]. We selected for the experiment only immature birds (i.e., hatched in the same calendar year) visiting this stop-over site for the first time in their life. We did not consider birds’ sex as both species are characterized by subtle sex dimorphism in morphological traits and only molecular sexing is a reliable method [28,29], which we did not apply in the present study.

**Figure 2:**
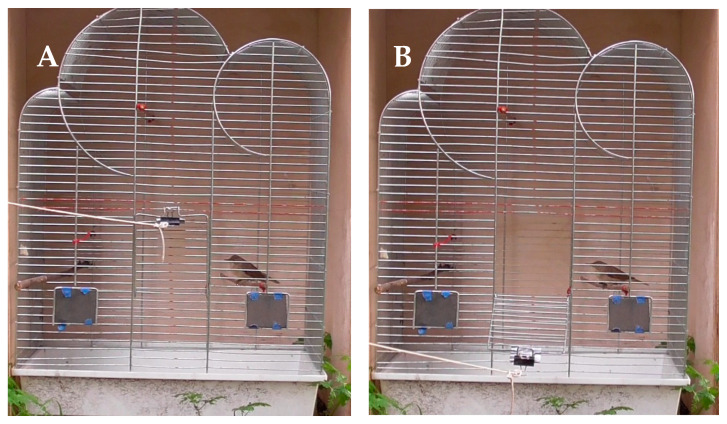
Experimental cage setting with the Reed Warbler before (**A**) and after (**B**) cage door opening.

Immediately after ringing and measuring we placed them into an experimental cage (commercial type, dimensions 52.5 cm × 32.5 cm × 72.0 cm; Figure 2). All the sides of the cage except for the front one with the door (dimension 13.0 cm × 13.0 cm) were covered (Figure 2) to minimize an effect of external stimuli that could affect the birds’ behaviour. A few-meters long string was attached to the door, allowing it to be opened from a distance without an interference during the experiment. Each bird was left in the closed cage for two minutes to adjust to the new environment, then the door was opened remotely by pulling the string (see example videos in Supplementary Materials). Then the bird was left to spontaneously escape the cage within two minutes. If that did not happen the bird was released by the observer. All the experiments were recorded with a commercial camera (Panasonic HC-V180, set up in 50 cm distance from the cage), thus, we further analysed video recordings to establish the occurrence and the timing of the spontaneous escape of each focal bird (see output file in Supplementary Data File). The experiments were performed from 8:00 to 11:00. In total, we tested 93 Sedge Warblers and 114 Reed Warblers.

We expected and could see on the videos that birds immediately after placement in the cage performed stress-induced behaviour but after few seconds they calmed down and started to explore the cage, evaluating the site. Based on that we assumed that while escaping the cage the main motivation of a bird was simply to leave an uninteresting/unfamiliar place, not to escape in panic, accepting any risk, even of being injured, just to save its life.

### 2.3. Statistical Analyses

To compare the proportion of individuals that escaped and did not escape from the experimental cage we used the chi-square test of independence. Then, we calculated the odds ratio. To investigate latency of escape from the cage in the group of individuals that escaped during the two minutes of the experiment we used the Cox proportional hazards model. The dependent variable was the number of seconds it took the bird to escape. We considered the birds that did not escape within two minutes as ‘censored’ observations (i.e., they were still in the data set when the observation time expired but had not yet escaped). The hazard function for each group represents the likelihood that a given bird of that species will escape the cage at a given time, assuming it is still in the cage at that time [30]. We checked the Cox proportional hazards model assumption that the hazard functions of the two groups are proportional across time (meaning that if one species was twice as likely to escape at 30 s, it would also be twice as likely to escape at 60 s, and so on). To visualize probability of staying birds in the cage we used a Kaplan-Meier plot of the survival curves for each species. We performed chi-square tests and calculated odds ratio in SAS version 9.4 (SAS Institute, Inc., Cary, NC). To plot the Kaplan-Meier survival curves we used *surviminer* package [30] in R software [31]. A level of significance α = 0.05 was used to asses statistical significance throughout.

## 3. Results

We found a significant association between species and whether the bird escapes the cage (chi-square test of independence, χ^2^_1_ = 5.56, *p* = 0.02). Sedge warblers escaped from the cage more frequently (41%) than Reed Warblers (25%; Table 1). The estimated relative risk was 1.61, with a 95% confidence interval being 1.08–2.39. This means that Sedge Warblers were 1.61 times more likely to escape the cage than Reed Warblers (95% confidence interval of 1.08–2.39).

**Figure 3:**
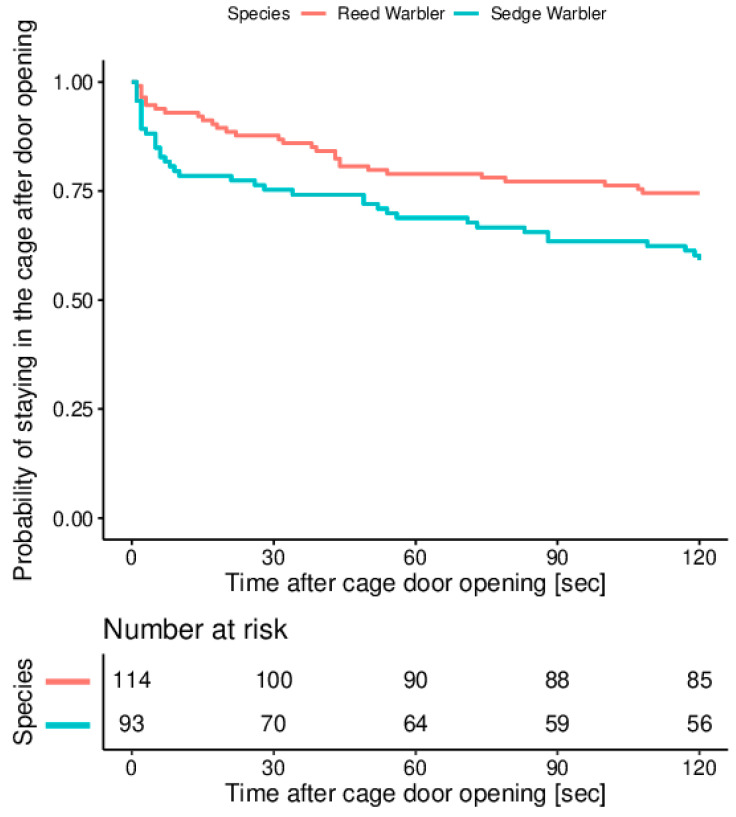
Kaplan-Meier survival curves for reed and Sedge Warblers tested during the autumn migration at the stop-over site at “Druzno Lake” reserve.

After 30 s with the cage door being opened, 88% (100 of 114 tested) of Reed Warblers and 75% of Sedge Warblers (70 of 93 tested) were still in the cage. At the end of experiment (i.e., after 120 sec after cage door opening), 75% of Reed Warblers and 59% of Sedge Warblers were still in the cage (Figure 3). The model’s assumption of proportional hazards was tested and found to be upheld (χ^2^_1_ = 0.27, *p* = 0.60), indicating that the results of the analysis may be reliably interpreted. We found a significant difference in hazard functions between sedge and Reed Warblers (χ^2^_1_ = 5.59, *p* = 0.02), which is expressed as a hazard ratio (the ratio of the two hazard functions with the hazard function for Sedge Warblers as the numerator and for Reed Warblers as the denominator). The estimated hazard ratio was 1.79 (95% CI: 1.11–2.91), indicating that at any given time, Sedge Warblers were 1.79 times more likely to escape from the cage compared to Reed Warblers.

## 4. Discussion

We found that 59% and 75% of tested individuals of sedge and Reed Warblers, respectively, did not escape from the cage during the two minutes of the experiment. This result is not surprising given a general behaviour of migratory birds, which in contrast to resident species are more likely to enter an unfamiliar environment, but once they are in, they are less explorative [21,32]. Migrants staging at each stop-over site for relatively short periods of time have to quickly familiarize with local food availability and predation risk [20]. Considering the costs and benefits of exploration, they may keep this to a minimum as they cannot use this information in the long term [33,34]. It has been found that migrants are less flexible in their behaviour (less explorative and less innovative) but have a better spatial memory compared to residents. This phenomenon has been attributed to a smaller forebrain, and an enlarged hippocampal formation in migrants [21]. It may explain why a relatively large proportion of tested individuals from both species did not find an escape route during the experiment period.

We expected Reed Warblers to leave the cage more readily than Sedge Warblers due to an apparent territorial behaviour of the former species at stop-over site that would predispose them to be more explorative in the cage. Contrary to our expectation, however, we found that Sedge Warblers were 1.6 times more likely to escape the cage than Reed Warblers. Moreover, in the group of escapers, Sedge Warblers escaped earlier/with lower latency after cage door opening compared to Reed Warblers. One possible explanation may be that even if Reed Warblers discover the opening faster than Sedge Warbler, they stay longer to explore the cage environment for possible suitability. On the other hand, Sedge Warblers may be escaping as soon as they discover the opening, being less bound to any migratory site. Another explanation may be linked to differences in foraging strategy at stop-over sites during the migration, which we did not consider *a priori* but in the context of the received results seems to have importance. There is evidence that Sedge Warblers, at least at some stop-over sites during autumn migration, search for one type of prey that is superabundant but only locally and periodically accessible, aphids (e.g., in France; [8,9,12]), while Reed Warblers forage on more common but variable prey items [8,9]. It has been found that specialized species pay attention to fewer cues than generalists which reduces heterogeneity in their environment [35] and speeds up decision-making [36,37]. Moreover, according to the dangerous niche hypothesis, specialists are less neophobic than generalists. Neophobia protects generalists against dangers encountered by them in various habitats [21,38]. In this context, one may expect that an aphid specialist, the Sedge Warbler, should pay attention to fewer cues than a generalist, the Reed Warbler. Thus, results of our experimental study could be interpreted that Sedge Warblers, owing to more focused perception of the environment, were able to perceive the open cage door and associate this event with the possibility to escape faster than Reed Warblers, which are usually more focused on multiple environmental cues.

Another explanation of the surprising species-difference in performance, not-excluding the one provided above, may be various reactions to the stress associated with handling [39]. It is possible that all the inter-species differences in behaviour and diet make the Sedge Warblers more resistant to stress situations. If so, Sedge Warblers could recover from the stress situation faster than Reed Warblers, and thus were able to perceive and process the information of the opening cage accordingly. This may be related to habituation factor. A two-minute habituation period may be too short for recovery of handling stress, especially if one species is more stress-resistant than the other. We were forced to keep both limited, the habituation and main experiment phases time, in order to avoid extended handling/waiting time for multiple birds being captured at the same time.

One might claim that birds placed in the cage were not expressing their actual cognitive ability-linked traits as they were simply frightened. They might be ready to accept any risk including injury to escape and save their life. However, our observations indicate that the majority of birds, after few second-long initial ‘hyperactive’ phase apparently calmed down (which we could call acclimatization) and started to explore the cage, hopping between the perches and sides of the cage or just sitting in the perch observing surroundings. It has been found that exploratory behaviour of wild-caught great tits (*Parus major*) in a novel environment was significantly repeatable [40]. Thus, we believe that both species-specific and individual cognitive abilities should be expressed even in unusual circumstances (experimental cage) and after a stressful situation (handling).

Our study being correlational has an inherent constraint. Our interpretation of the revealed inter-species difference in the escape behaviour, even if plausible and parsimonious, may not necessarily be related to the attributed species differences in migration strategy, diet, and/or cognitive abilities. More studies including various traits of birds (e.g., sex, body size, body mass, initial stress level) are needed to fully understand factors affecting birds’ decisions about escaping/not escaping an experimental cage. Nevertheless, the observed differences between the sedge and Reed Warblers are ground solid and indicate that the behavioural traits may evolve differently in closely related species of very similar ecology. Besides, the study focusing on wild migrant species provides valuable insight into cognitive abilities-linked traits of this ecological group.

## 5. Conclusions

We found inter-species differences in the cage escape behaviour between two closely related warbler species. We attribute the found differences to various cognitive abilities-linked traits which may have evolved together with different refuelling strategies at stop-over sites expressed in generalist versus specialized diet, and non-territorial versus territorial habitat use in Sedge Warblers and Reed Warblers, respectively. Understanding a relationship between cognitive abilities-linked traits and migration strategies in birds is important to comprehend reactions of organisms to rapidly changing environments. For instance, relying on memory in combination with little explorative abilities and high avoidance of changes in the familiar environment leaves migrants with only little room to respond to unpredictable environmental change [6]. Considering carry-over effects in migratory birds’ [41,42] cognitive abilities-driven reaction to environmental changes at stop-over sites may have serious consequences at other stages of avian annual life cycle. Thus, we encourage the study of cognitive abilities-linked traits in various wild species. Only by having a wider perspective will we be able to fully understand the evolutionary processes behind animal cognition.

## Figures and Tables

**Table 1:** Numbers of sedge and Reed Warblers that escaped and not escaped from the experimental cage within two minutes after door opening.

Species	Escaped	Non-Escaped	Total
Sedge Warbler	38 (41%)	55 (59%)	93 (100%)
Reed Warbler	29 (25%)	85 (75%)	114 (100%)
Total	67	140	207

## Data Availability

The dataset supporting this article has been uploaded as part of the electronic supplementary material.

## Supplementary Materials

The following are available online at https://www.mdpi.com/2076-2615/11/3/639/s1, File S1: Supplementary Movie with two experiments, File S2: Supplementary Data File with results of all experiments.
